# Solid‐State Vortex Laser at Eye‐Safe Band: A Perspective

**DOI:** 10.1002/nap2.70006

**Published:** 2026-01-28

**Authors:** Haojun Zhang, Zhichao Zhang, Lihong Hu, Chunqing Gao, Shiyao Fu

**Affiliations:** ^1^ School of Optics and Photonics Beijing Institute of Technology Beijing China; ^2^ Key Laboratory of Information Photonics Technology Ministry of Industry and Information Technology of the People's Republic of China Beijing China; ^3^ Key Laboratory of Photoelectronic Imaging Technology and System Ministry of Education of the People's Republic of China Beijing China; ^4^ National Key Laboratory on Near‐Surface Detection Beijing China

**Keywords:** eye‐safe band, solid‐state laser, structured beams, vortex laser

## Abstract

Vortex beams, known for carrying orbital angular momentum (OAM), demonstrate significant potential across LiDAR, laser communications, high‐precision metrology, imaging, and quantum information. Generating vortex beam on demand is the basis and crucial for above implementations. Recently, solid‐state vortex laser in eye‐safe band have attracted considerable attention and been regarded as one of ideal sources for vortex beam generation, because of its advantages in high mode purity, mode selectivity, high output power, and locating in atmosphere window. In this perspective, we summarize the schemes of solid‐state vortex lasers in eye‐safe band, survey their potential in multi‐functional LiDAR systems, and discuss prospects for future development.

## Graphical abstract

As one of the ideal vortex sources, solid‐state vortex laser at eye‐safe band has attracted considerable attention because of its potentials in multifunctional lidar. In this perspective, we summarize the schemes of eye‐safe vortex laser and discuss prospects for future development.

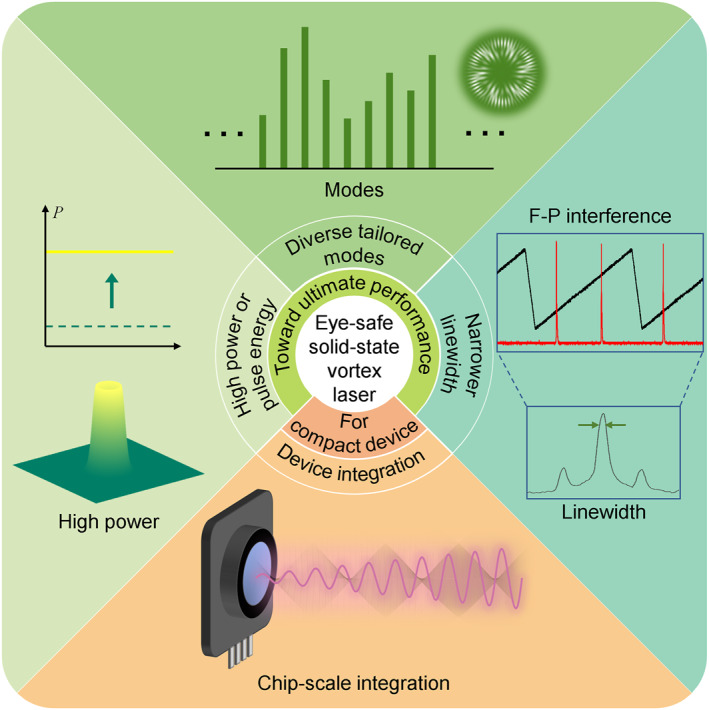

## 1 Introduction

Vortex beams are a kind of topological structured beams characterized by a helical wavefront exp (−i*lϕ*), with the integer *l* the topological charge and *ϕ* the azimuthal angle. An amazing feature of vortex beams is that, each photon in them carries orbital angular momentum (OAM) of *l*ℏ, with the ℏ reduced Plank constant [1]. Different OAM mode are orthogonal with each other, thereby forming an infinite‐dimensional Hilbert space. This attractive characteristic of vortex beams contributes to lots of state‐of‐art scenarios, such as rotational Doppler LiDAR [2], optical communication [3], optical tweezers [4], laser processing [5], and quantum information [6, 7].

However, the practical implementations of vortex beams rely on efficient and reliable generation schemes. Current techniques can be broadly categorized into intra‐cavity and extra‐cavity approaches. Extra‐cavity approaches, such as spatial light modulators (SLMs) [8], spiral phase plates (SPPs) [9], and forked gratings [10], offer flexibility but often suffer from limitations including low conversion efficiency, low damage threshold, poor mode purity, and system complexity, making them unsuitable for engineering. Consequently, vortex lasers that directly output vortex beams from the laser resonator have become a research focus because of their compact structure, high efficiency and low cost. Solid‐state vortex lasers are particularly preferred, exhibiting high mode purity, selective topological charge, and high energy output.

Leveraging gain media and nonlinear conversion, solid‐state vortex lasers offer flexible wavelength selection capabilities. Significant attention has been focused on the eye‐safe band (typically 1.4–2.2 μm) due to its overlap with operational windows in several potential application domains for vortex beams [11]. Taking rotational Doppler LiDAR as an example, this band not only offers higher permissible exposure limits and minimal disruption on daily life but covers atmospheric transmission windows and absorption lines of various atmospheric constituents, thereby facilitating the measurement of angular motion and analysis of atmospheric eddy.

In this paper, we focus specifically on solid‐state vortex lasers operating within the eye‐safe band. In Section 2, we discuss various schemes of solid‐state vortex beams. Then, two primary approaches for achieving vortex laser output in the eye‐safe band are discussed in Section 3. One utilizing direct oscillation from rare‐earth‐ion‐doped gain media, and the other employing nonlinear frequency conversion. In Section 4, we discuss the application prospects of eye‐safe vortex lasers in novel LiDAR detection systems. Finally, in Section 5, we outline our views on future development directions for eye‐safe solid‐state vortex lasers.

## 2 Schemes of Solid‐State Vortex Laser

The Laguerre–Gaussian (LG) modes, as a kind of typical vortex modes, are Eigen solutions of Helmholtz equation in cylindrically symmetrical laser resonator, thus enabling direct oscillation. The key to oscillate LG modes is to suppress the fundamental Gaussian lasing via selective gain‐loss control. In this way, several schemes have been developed for direct generation of vortex beams in solid‐state regime.

### 2.1 Annular Pumping

Annular pumping of an end‐pumped solid‐state laser has emerged as the most direct and efficient scheme for vortex laser. By tailoring the pump beam into a ring‐shaped intensity profile that overlaps optimally with the transverse distribution of LG modes, the gain of desired mode is selectively enhanced, thereby suppressing competing fundamental Gaussian oscillation. So far, annular pumping is commonly implemented using optical fibers [12, 13], a hollow mirror [14, 15], a diffractive optical device [16, 17, 18, 19], a cone lens [20], etc.

**FIGURE 1:**
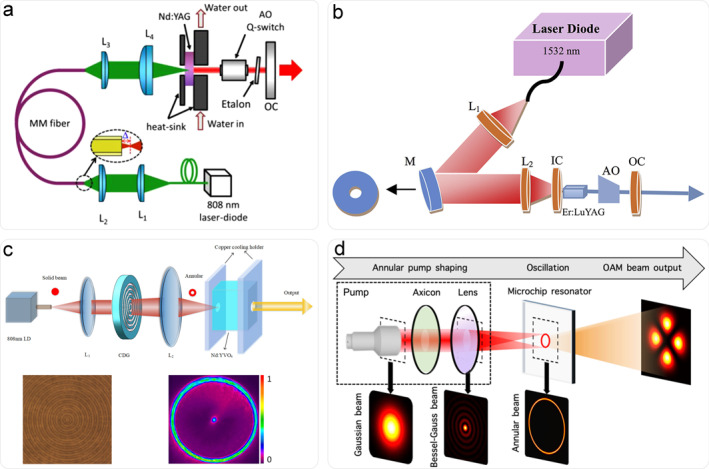
Annular pumping schemes enable solid state vortex laser. (a) Defocused optical fiber [12]. (b) Hollow mirror [15]. (c) CDG [18]. (d) Axicon [20]. (a) Reprinted from Ref. [12] with permission. Copyright 2017 Elsevier. (b) Reprinted from Ref. [15] with permission. Copyright 2015 Elsevier. (c) Reprinted from Ref. [18], under the terms of the CC BY 4.0 license. Copyright 2024 The Author(s). (d) Reprinted from Ref. [20] with permission. Copyright 2024 Optica Publishing Group.

Optical fibers are effectively employed for generating annular pumps since early years. As illustrated in Figure 1a, annular beams are generated by coupling a defocused Gaussian beam into a multi‐mode optical fiber [12]. By adjusting the defocusing length, diameter and the type of fiber, a range of different pumping intensity distributions could be obtained, thus enabling flexible manipulation of output vortex beams. Pure LG_0,*l*_ mode with the highest topological charge of 23 has been obtained. And the beams' polarization and chirality can be directly controlled by tilting the fiber coupler [13]. However, pump energy is severely constrained by the low damage threshold of coupled‐fibers, adversely affecting vortex beams' oscillation and output. To achieve annular pumping in free‐space, hollow mirrors are used [14, 15], as shown in Figure 1b. Although the pump energy is no longer constrained compared to the fiber‐coupled scheme, the hollow mirror design intrinsically incurs significant pumping losses, degrading the quantum efficiency.

Diffractive optical devices offer another route for generating annular pump beams. The earliest implementation, reported in 2005, used circular aperture diffraction to create an annular pump profile [16]. However, the conversion efficiency remained modest. To overcome this limitation, circular Dammann gratings (CDGs)—radial phase gratings that diffract an incident Gaussian beam into equal‐intensity concentric rings—were introduced [17, 18] as shown in Figure 1c. For flexible generation of vortex beams, a programmable diffractive device, namely, the digital micromirror device (DMD), is developed and proposed for pump beam shaping. In this means, the gain distributions could be controlled flexibly in the laser cavity because of the dynamic pump shaping. Therefore, various high‐order transverse modes including vortex modes are switchable outputted from the resonator. For instance, the Hermite–Gaussian (HG) modes HG_01_ and HG_02_, LG modes LG_0,1_ and LG_0,2_ and vortex array beam formed by superposition of different modes have been successfully generated by loading specific patterns on DMD [19].

In addition, the stable annular pumping scheme is also achieved through an axicon associated with a lens. Figure 1d shows a microchip Nd: YVO_4_ laser scheme. When a Gaussian pump beam incidents in the axicon, it diffracts to the Bessel–Gauss (BG) beam in the near field. Then, the convex lens placed in the Bessel region transformed the BG beams into the annular shaped perfect vortex in the focal plane. By shifting the excitation position of the microchip, the width and radius of the input annular pump can be adjusted, thereby switching the oscillation modes. Our group has recently achieved single‐frequency LG_0,±1_ and LG_0,±2_ vortex beam output from this scheme [21].

### 2.2 Off‐Axis Pumping

Off‐axis pumping has also become one of the most important ways to generate vortex beams in solid‐state lasers. Common off‐axis pumped lasers excites HG modes, which need extra‐cavity conversion devices to transform into vortex beams. Notably, recent studies have demonstrated that novel off‐axis pumped schemes can directly generate LG beams, such as rotating‐induced off‐axis pumping [22], off‐axis “optical needle” pumping 23, and off‐axis pumped non‐planar‐ring‐resonator (NPRO) [23]. By adjusting the relative position between the pump beam and the optical axis of the laser cavity, the high‐order transverse mode can be effectively excited, and the LG mode can be directly output. It has been reported that high‐order LG modes ranging from LG_0,0_ to LG_0,+30_ were obtained via off‐axis pumped Nd:YAG NPRO laser [24]. Because additional intracavity elements are avoided, the laser scheme with off‐axis pumping also reduces complexity and cost, and preserves robustness.

### 2.3 Defect Mirror

The aforementioned schemes primarily enhance the gain of specific LG modes, enabling to outcompete the fundamental Gaussian mode oscillation. Unlike the above schemes, using a defect mirror in a solid‐state laser is to suppress the fundamental mode oscillation by introducing intentionally manufactured damage points on the cavity mirror while allowing the oscillation of high‐order transverse modes [25]. The scheme requires a proper matching between the defect size and the transverse mode distribution, then enabling extremely high‐order vortex modes lasing. By precisely designing the ratio of the radius of the circular pattern on the defect mirror to the radius of the transverse laser mode, a clear theoretical design criterion is formed, and a vortex beam output with topological charge up to 288 has been reported in a Nd:YVO_4_ laser [26]. It significantly improves the upper limit of the order of the vortex laser and verifies the good controllability and stability of the scheme. In addition, the intra‐cavity defect mirror schemes have expanded beyond linear cavities to multi‐folded novel laser configurations [27], and also shown good adaptability in the field of ultrafast lasers [28].

Whereas defect mirror schemes enable more precise mode selection, the central damaged design wastes unused pump energy in the core region. The pump inefficiency undoubtfully elevates the oscillation threshold for vortex modes. Therefore, integrating defect mirrors with pump‐shaping schemes could be considered to optimize energy utilization in future implementations.

### 2.4 Intra‐Cavity Modulation Elements

Unlike gain‐loss control mechanism, inserting modulation devices into a solid‐state laser is also an efficient way to generate vortex beams through intra‐cavity phase and polarization modulation. Modulation devices include SLM, Q‐plate (QP), metasurfaces, and others. Under this configuration, vortex beams with high purity, high efficiency, and high topological charge could be output, also showing the advantages of dynamic adjustment and multi‐mode switching.

**FIGURE 2:**
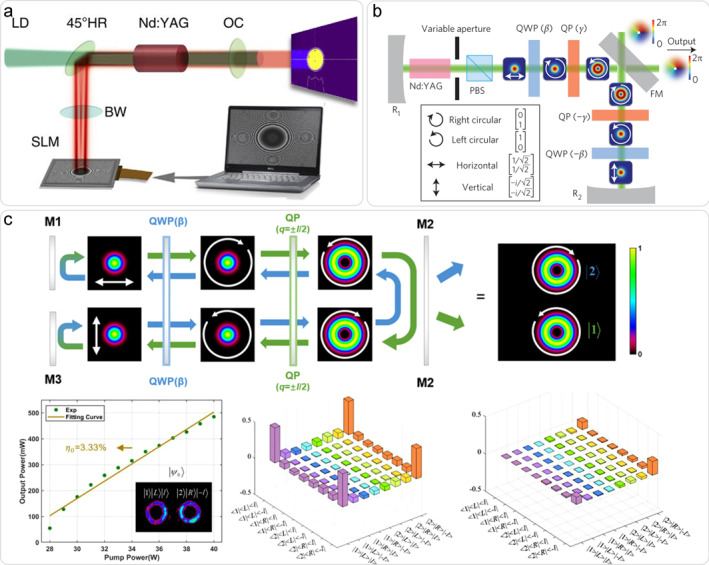
Vortex lasers through intra‐cavity mode modulation. (a) The digital laser [29]. (b) A QP‐inserted HOP laser [30]. (c) A classical GHZ‐like non‐separable state laser [31]. (a) Reprinted from Ref. [29] with permission. Copyright 2013 Springer Nature. (b) Reprinted from Ref. [30] with permission. Copyright 2016 Springer Nature. (c) Reprinted from Ref. [31] with permission. Copyright 2023 John Wiley and Sons.

The concept of digital laser has opened up a new paradigm for the digital control of intracavity laser modes [29]. Figure 2a depicts a typical digital laser, where a SLM is employed as an end mirror. By generating digital holograms and encoding them on a phase‐only SLM, the on‐demand output such as HG, LG, Airy, and other modes are realized. In order to select the LG mode in the cavity, a high‐loss annular aperture with a phase–only radius of curvature is comprised as the digital hologram. In this way, the topological charge of vortex beam can be switched without additional adjustment of the cavity but changing the encoded holograms. To enhance the spatial resolution of the output and achieve precise control over its profile, 4‐*f* telescopes have been integrated into degenerate cavity lasers [32, 33]. This scheme demonstrates considerable potential for generating high‐order vortex beams. Furthermore, the incorporation of conjugate SLMs in a ring cavity allows on‐demand formation of multi‐OAM‐mode sources, offering new opportunities for fundamentals and applications involving high‐dimensional OAM modes [34]. However, the digital laser is still limited by the low damage threshold and intrinsic diffraction.

QP is a kind of spatially varying wave plate based on liquid crystal material [35]. As a spin–orbit angular momentum coupling device, it has been widely used in various solid‐state lasers. The core design objective of such scheme is to achieve the intra‐cavity mode self‐consistency. Back in 2016, a mode‐selective vector vortex laser was reported [30]. As shown in Figure 2b, a pair of quarter‐wave plates (QWP) and QPs were used to form a self‐consistent mode conversion scheme, and the on‐demand generation of vortex mode and cylindrical vector beam (CVB) on high‐order Poincaré sphere (HOP) was realized with high mode purity. Following such idea, a classical Greenberger‐Horne‐Zeilinger (GHZ)‐like non‐separable state is illustrated to be output from a special designed folded resonator with a single QP and QWP, as shown in Figure 2c [31]. Three degrees‐of‐freedom (DoFs) as OAM, SAM, and wave vector could be tailored well to form a non‐separable classical state, which is very similar with that of GHZ entanglement in quantum physics, providing an effective classical tool for study on complicated quantum process. Besides, in order to further improve the flexibility of mode control, the “order switchable Q‐plate (OSVWP)” scheme is also proposed [36]. By cascading the first‐order and third‐order QPs in the cavity and adding two controllable QWPs, the order addition and subtraction operation is realized. The second‐order or fourth‐order CVB and vortex beams can be output on demand in the same Nd:YAG laser with high mode purity. Compared to digital lasers, the damage threshold and the transmittance loss of QP‐inserted lasers are largely improved.

Compared with the common QP, the metasurface not only shows mechanisms of SAM‐OAM coupling but also achieves high‐order asymmetric vector‐controlled output mode by designing the structural parameters. Metasurface is a two‐dimensional device arranged by micro‐nano structures [37]. It is usually a sub‐wavelength structure, enabling it to be highly integrated into a micro‐optical system, which significantly improves the compactness of the device. The metasurface structure based on all‐dielectric materials can break the symmetry constraint between spin and orbital angular momentum [38]. Thus, vortex beams with complex wavefronts are hopeful to be produced on chip. As reported in 2020, a vortex laser exploiting J‐plate, a metasurface, achieves vortex beam output with topological charges of up to 100, and supports simultaneous lasing of asymmetric OAM superposed state.

### 2.5 Other Schemes

In addition to the widely used schemes, other attempts have also been employed to obtain vortex beams in solid‐state lasers.

The thermal lensing effect is often regarded as an unfavorable factor in gain medium, but it has been gradually transformed into an effective mechanism for generating high power vortex beams. The refractive index change caused by the pump‐induced thermal gradient could be employed to achieve high‐order LG mode oscillation. By adjusting the pump spot size and the grazing incidence rebound angle, the gain region can be highly circularly symmetrical on the cross section, thereby inducing a thermal lens with an approximate spherical surface and a negative spherical aberration [39]. This spherical aberration makes the fundamental mode unstable at thermal stability boundaries, whereas the LG_0,1_ mode with a wider spatial distribution selectively oscillates.

**FIGURE 3:**
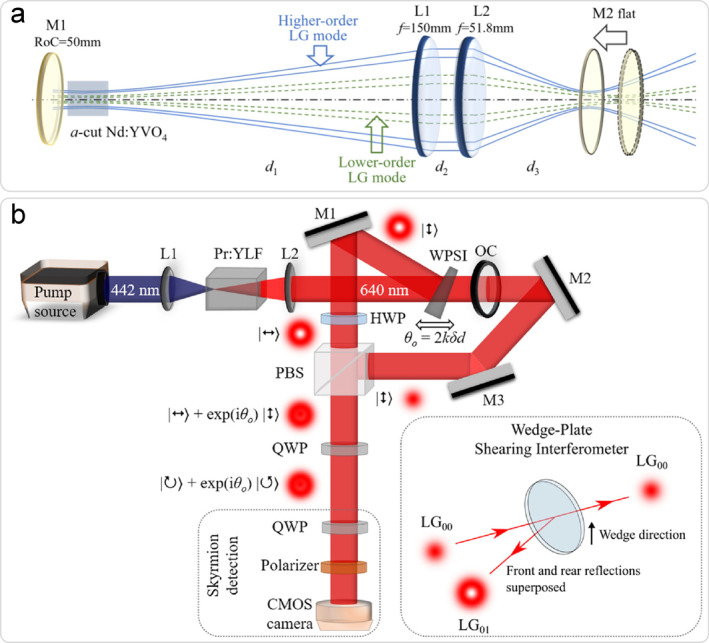
Vortex lasers with unconventional configurations. (a) A SA‐induced vortex laser [42]. (b) An optical skyrmions laser using WPSI [43]. (a) Reprinted from Ref. [42] with permission. Copyright 2023 John Wiley and Sons. (b) Reprinted from Ref. [43] with permission. Copyright 2024 Optica Publishing Group.

Another interesting scheme is to introduce controllable spherical aberration (SA) into the cavity, which enhances the gain difference between different transverse modes and promotes the selection of high‐order LG modes [40]. Furthermore, through appropriate choice of the focal length of the intracavity lens, this configuration enables the excitation of LG modes with non‐zero radial indices [41]. The experimental arrangement is depicted in Figure 3a. With the improvement of intracavity lens system, the selective generation of LG modes has achieved more and more wide switchable range as LG_0,±27_ to LG_0,±317_ [42], which represents the highest‐order vortex laser reported to date. Also, in the visible band, a 640 nm Pr:YLF laser exploiting intracavity SA manipulation has been reported, with switchable topological charges from 12 to 42 [44].

In addition, a novel laser scheme based on interferometric mode‐converting output couplers have also emerged in recent years. By introducing an output coupler like wedge‐plate shearing interferometer (WPSI) into the cavity, the oscillating Gaussian mode is directly converted into vortex mode output [45]. However, the high‐order vortex mode can be barely achieved. Following this idea, an optical skyrmion laser [43] is constructed, as shown in Figure 3b, in which high‐purity optical skyrmions are generated in the visible light band. Notably, the scheme exhibits three key advantages: structural compactness, robust stability, and efficient power scaling capability.

## 3 Implementation at Eye‐Safe Band

The eye‐safe wavelength band (typically 1.5–2.0 μm) is a precious spectral resource for laser communication and detection. There are many ways reported already to obtain such bands such as the mature doped fiber [46, 47]. In the solid‐state regime, beams located at eye‐safe band are usually generated through two means: direct oscillation through rare‐earth‐ions working in these bands, or nonlinear frequency conversion.

### 3.1 Direct Oscillation

The laser output in the eye‐safe wavelength band is chiefly contingent on solid‐state crystal or ceramic doped with erbium (Er^3+^, ∼1.6 μm band), thulium (Tm^3+^, ∼1.9 μm band), and holmium (Ho^3+^, ∼2.1 μm band).

The primary emission cross‐sections of Er^3+^‐doped lasers in the eye‐safe band are at 1617 and 1645 nm, corresponding to communication bands and atmospheric windows, and the absorption cross‐sections are around 1470 and 1532 nm [48, 49]. The 1470 nm region features multiple absorption peaks across a broad spectral range, making it suitable for pumping with cost‐effective laser diodes (LDs) despite their inherent wavelength instability. In contrast, the narrower absorption peak around 1532 nm demands fiber lasers with greater wavelength stability as pump sources. The output wavelength of Er‐doped lasers is primarily governed by the upper‐level population density, with reabsorption effects generally favoring 1645 nm emission. Compared to Nd‐doped lasers, which are typically four‐level systems, eye‐safe Er‐doped lasers are considered quasi‐three‐level systems with lower energy conversion efficiency. The additional intracavity losses introduced by the modulation devices when generating vortex beams further raise the lasing threshold, presenting significant challenges in the design of Er‐doped solid‐state vortex lasers. Nevertheless, Er‐doped gain media still offer the most comprehensive technical routes among the three major rare‐earth ions.

**FIGURE 4:**
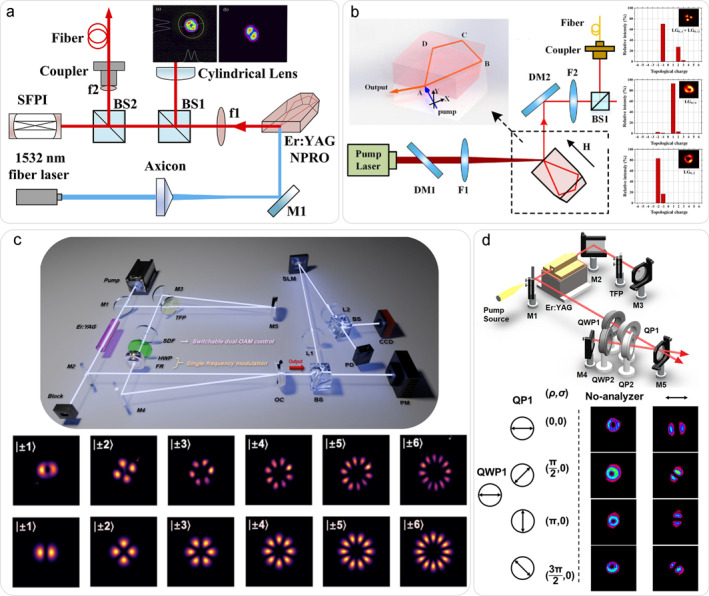
Er:YAG vortex lasers constructed by different schemes. (a) Annular pumping [51]; (b) off‐axis pumping [52]; (c) defect mirror [53]; (d) intra‐cavity modulation elements [54]. (a) Reprinted from Ref. [51] with permission. Copyright 2023 Optica Publishing Group. (b) Reprinted from Ref. [52] with permission. Copyright 2025 Optica Publishing Group. (c) Reprinted from Ref. [53] with permission. Copyright 2025 Optica Publishing Group. (d) Reprinted from Ref. [54] with permission. Copyright 2020 Optica Publishing Group.

In annular pumping configurations, early approaches utilized 50‐cm‐long capillary fibers to transform Gaussian beams into annular profiles, enabling high‐energy high‐mode‐purity first‐order vortex laser. This approach imposes high demands on both system size and precision [50]. Figure 4a illustrates an alternative scheme employing axicon‐generated annular pumping in conjunction with a NPRO, constituting a single‐frequency vortex laser at 1645 nm. The achieved narrow linewidth of 6 kHz demonstrates potential for LiDAR [51]. The NPRO can also be integrated with off‐axis pumping to generate high‐power, single‐frequency, and chirality‐switchable vortex beams with orders ranging from −2 to 2, as shown in Figure 4b [52]. However, compared to Nd:YAG NPRO lasers, Er^3+^ systems are constrained by their smaller emission cross‐sections, resulting in relatively lower maximum achievable topological charges. To generate higher‐order vortex beams with flexible tunability, defect mirrors have been incorporated into Er:YAG laser systems. By employing a unidirectional ring cavity design with an adjustable defect aperture, a single‐frequency dual‐OAM mode output ranging from ± 1 to ± 6 can be achieved, as shown in Figure 4c [53]. Figure 4d shows vortex laser generated by intracavity modulators. By adding a pair of QPs and QWPs, all cylindrical vector beams on a single HOP can be obtained [54].

**FIGURE 5:**
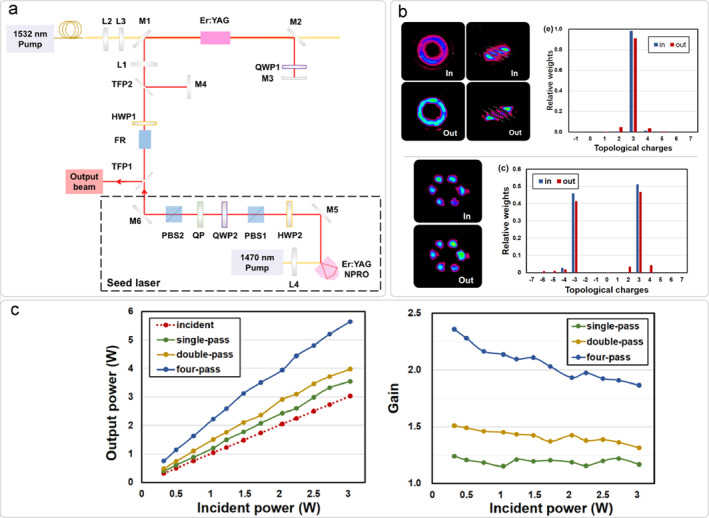
Four‐pass Er:YAG vortex MOPA [55]. (a) Configuration of the system. (b) Intensity distributions and mode purity before and after passing through MOPA. (c) Power amplification performance for different pass case. Reprinted from Ref. [55] with permission. Copyright 2023 Optica Publishing Group.

Furthermore, master oscillator power amplifier (MOPA) technology has been demonstrated to be efficacious in this band. Figure 5 shows a four‐pass Er:YAG vortex MOPA system [55]. This scheme achieves high mode purity and high gain of amplified vortex beams, showing good usability in both single‐mode and dual‐mode vortex beams, providing a model for the application of MOPA technology in other media.

Tm‐doped gain media exhibits a broader emission spectrum spanning 1.9–2.0 μm [56]. Typically, 790 nm LD is used for pumping, forming a quasi‐three‐level structure. Compared to Er^3+^ systems, the presence of cross‐relaxation processes in Tm^3+^ pumping enables higher quantum efficiency, making it an ideal candidate for developing highly efficient, broadly tunable eye‐safe lasers around 2.0 μm. However, because of the high energy and the scarcity of devices in the corresponding wavelength band, intra‐cavity modulation methods are less commonly used. Most lasers rely on annular pumping and off‐axis pumping, with research primarily focused on tunable and pulsed vortex beams. In terms of tunable laser, by employing off‐axis pumping and a F–P etalon, wavelength tuning with a bandwidth of 39 nm is achieved for vortex beams up to the 16th order, which have potential application in high‐capacity optical communication involving wavelength and OAM‐MDM [57]. For lasers operating in pulsed regime, high material damage thresholds are always required. Thus, off‐axis pumped solid‐state laser is generally the only method used, enabling the generation of about 100 fs ultrashort pulses [58]. This scheme can be employed to produce pulsed vortex beams in the mid‐infrared region. However, Tm‐doped media are generally not used for MOPA, as their thermal conductivity is insufficient to maintain the mode purity of vortex beams.

The Ho^3+^‐doped laser operating at room temperature is a quasi‐two‐level laser system, with identical pump final level and the laser upper level [59]. The laser final level and the ground state level are also the same, leading to a smaller quantum defect. Throughout the process of photon formation, there is no relaxation process between the pump final state level and the laser upper level, thereby reducing the pump energy loss caused by this process. These intrinsic properties enable Ho‐doped lasers operating in the quasi‐two‐level scheme to achieve high conversion efficiency with reduced thermal loading. The absorption peak of Ho‐doped media lies near 1910 nm, and typically pumped by Tm‐doped lasers, whereas emission peaks emerge around 2.1 μm, notably at 2090, 2097, and 2122 nm. This band coincides with the significant absorption peak of water molecules, making it indispensable in applications such as wind‐measuring LiDAR and laser medicine. In the field of vortex lasers, Ho‐doped media are more commonly used in MOPA systems rather than in directly constructing vortex lasers. After single passing through a rod of Ho:YAG, beams with multi‐OAM modes can be amplified, with the gain ratio being affected by the positioning of the signal and pump light. For a three‐lobed vortex case, the maximum gain can reach 8.8 [60].

### 3.2 Non‐Linear Conversion

Nonlinear optical techniques offer a flexible and powerful route for the generating vortex beams at eye‐safe band. Compared with direct oscillating inside a laser cavity, non‐linear frequency conversion is to transform vortex beams from their original wavelength into eye‐safe band. Optical parametric oscillations (OPO) and stimulated Raman scattering (SRS), are usually considered as an effective nonlinear means in solid‐state regime.

Optical parametric processes are a difference frequency of three‐wave mixing that allow the energy transportation among pump beams (*ω*_p_, *k*_p_), signal beams (*ω*_s_, *k*_s_) and idler beams (*ω*_i_, *k*_i_), satisfying energy conservation (*ω*_p_ = *ω*_s_ + *ω*_i_), and momentum conservation (*k*_p_ = *k*_s_ + *k*_i_). This process typically requires the simultaneous input of pump and signal wave to achieve signal amplification. In contrast, OPO introduces the optical parametric process into a resonator, eliminating the need for external signal injection while splitting the pump wave directly into signal and idler components. The wavelengths of the signal and idler are primarily determined by the nonlinear crystal's phase‐matching conditions, offering broad wavelength tunability and flexible adjustability. The presence of the resonator also significantly enhances the conversion efficiency of the OPO, enabling high‐power/energy output.

For vortex beams generation via OPO, the pump beams can be either a vortex beam or a Gaussian beam. Consider an OPO system equipped with a 1064‐nm vortex pump beam. The OAM of the pump beams is usually completely transferred to the signal output, whereas the idler output is a fundamental Gaussian mode. The wavelengths of both beams can be tuned by adjusting the angle of the nonlinear crystal, with the signal wavelength ranging from 1953 to 2158 nm [61]. In the case of a Gaussian beam pump, OPO has been shown to generate vortex beams of the same order in two different wavelengths, in a manner similar to the off‐axis pumping method in vortex lasers [62].

**FIGURE 6:**
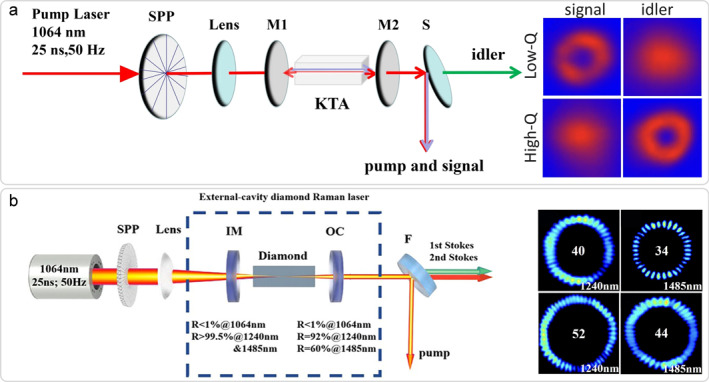
Eye‐safe solid‐state vortex laser from non‐linear conversion. (a) A KTA optical vortex parametric oscillator and its output in different cavity configuration [63]; (b) Optical vortex pumped diamond Raman laser and its vortex outputs across different cavity configurations and Stokes orders. [64]. (a) Reprinted from Ref. [63], under the terms of the CC BY 4.0 license. Copyright 2021 The Author(s). (b) Reprinted from Ref. [64], under the terms of the CC BY license. Copyright 2024 The Author(s).

For the non‐critical phase matching (NCPM), the primary advantage lies in the absence of walk‐off effects and the favorable mode‐overlapping condition, rendering it particularly suitable for high‐power pumping. When vortex beams are used for pumping, the OAM in the signal/idler beams can be modulated by adjusting the cavity length or the *Q* factor. As shown in Figure 6a, it has been employed to realize mJ‐level mid‐infrared vortex light output, which can be in either single or multiple OAM states [63]. The setup emits signal wave around 1.5 μm, with the corresponding idler wave located in the vicinity of 3.5 μm. Furthermore, other schemes such as QP can be also used in oscillations to achieve vortex output [65].

SRS is a third‐order nonlinear effect. Under high‐intensity pump illumination, the oscillating electric field induces periodic modulation of the molecular polarizability in the medium, resulting in a frequency shift in a portion of the output. This shift corresponds precisely to the vibrational or rotational frequency of the molecules, known as a Stokes shift. This process can undergo multiple SRS events on a single photon, thereby generating *n*th order Stokes light. Among solid‐state media, diamond stands out because of its remarkably high Raman gain coefficient, exceptional thermal conductivity, and extensive transmission spectrum, making it a crucial material for SRS. It can efficiently convert the well‐established 1064 nm laser to the 1.5 μm band through cascaded SRS, offering the potential for high‐power high‐mode‐purity eye‐safe lasers [66]. The order of vortex beams generated by SRS lasers is influenced by the pump beam and the resonator configuration. By combining SRS with off‐axis pumping, a first‐order vortex beam with up to 22 W output power has been demonstrated [67]. Furthermore, when a first‐order vortex beam is employed as the pump source and a confocal‐symmetric cavity is utilized, as shown in Figure 6b, vortex outputs of up to ± 26th order at the first Stokes level and up to ± 22nd order at the second Stokes level (1485 nm) have been achieved [64].

## 4 Applications of Eye‐Safe Solid‐State Vortex Laser: Multi‐Functional LiDAR

For a common LiDAR, the distance and radial velocity of a target are acquired by measuring the time of flight (ToF) and Doppler shift of light. At eye‐safe band, conventional solid‐state LiDARs have been primarily applied to wind measurement. Based on the linear Doppler effect, such systems operating around 1.6 or 2 μm enable high‐precision detection of wind speed and atmospheric composition [68, 69]. The sources for these applications typically emphasize single longitudinal mode operation, high pulse energy, and a sufficiently wide pulse width. However, conventional LiDAR is incapable of detecting angular motion. Vortex beams carry OAM, thus provide a new DoF for bring more functions for LiDARs. Especially, the rapid development of eye‐safe solid‐state vortex laser provides an ideal source to construct multi‐functional LiDAR. The advent of the rotational Doppler effect (RDE) has engendered a novel dimension for LiDAR. The fundamental principle is that when a vortex beam carrying OAM (with a topological charge of *l*) illuminates an object rotating around the beam axis at an angular velocity Ω, the reflected or scattered light will produce a frequency shift of Δ*f* = *l*Ω/2π [2]. By measuring the frequency shift with high precision, the angular velocity of the target can be inferred directly. It has opened up a new technical pathway for identifying and characterizing rotating targets. To implement RDE in practical detection, a variety of technical solutions have been developed. For instance, the advances of vortex mode decomposition for echo signals at the receiver could decouple specific OAM modes the so as obtaining RDE shift [70, 71]. This method maximizes the utilization of the echo signals from LiDAR and can be applied to upgrade existing LiDAR systems.

However, to fully exploit the RDE, it is necessary to tailor the output probing beams for new types of LiDAR systems, which requires novel vortex laser sources. Extensive research has been conducted on RDE manifestations using various structured beams. Specifically, vectorially structured light with spatially variant polarization resolves directional ambiguity in rotational motion [72], whereas multi‐OAM beams effectively suppress the broadening of the RDE spectrum introduced by misalignment between the probe beam and the target's rotation axis, which enhances the measurement accuracy of angular motion [73]. These findings demonstrate that optimizing beam structures represents a crucial development direction for rotational Doppler LiDAR systems.

Moreover, the application of the RDE is not limited to solid hard targets but has also been extended into fluids to measure its flow vorticity [74]. Taking the detection and characterization of air vortices as an example, superimposed vortex beams carrying opposite topological charges can be used as the probing beam and directed into the air vortex. By analyzing the beat frequency signal of the scattered beam, the internal angular velocity of the air vortex can be laterally mapped [75]. This technique can be applied to the detection of aviation safety hazards such as aircraft wake vortices and serves as a model for the application of the RDE in wind fields, atmospheric turbulence [76], and underwater vortices [77], thereby expanding the application scope of vortex beams.

## 5 Outlook

In this paper, we have overviewed recent advances of solid‐state vortex lasers at eye‐safe band, which covers the basic principles of various vortex lasers, specific their technical implementation, and prospective application scenarios. In terms of vortex beam generation, we comb through the development from gain control schemes such as annular pumping and off‐axis pumping to loss control schemes like defective mirrors and intra‐cavity modulation elements. These schemes make it possible to achieve high‐order, high‐power, and high‐mode‐purity vortex beams in both continuous‐wave and pulsed operations. Regarding the implementation at eye‐safe band, direct generation schemes utilizing Er^3+^, Tm^3+^, and Ho^3+^ doped gain media, as well as nonlinear frequency conversion including OPO and SRS, are discussed. In terms of expectation of future applications, we convince the unique physical properties of vortex beams endow them with great engineering potential in domains as multi‐functional LiDAR.

**FIGURE 7:**
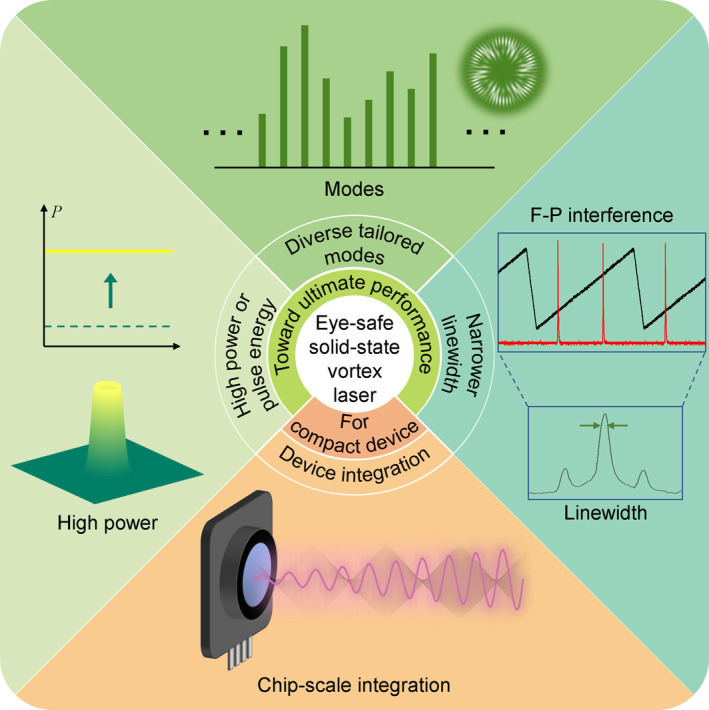
Future perspectives of eye‐safe solid‐state vortex laser.

Looking ahead, in our view, the future development of eye‐safe solid‐state vortex laser may toward following directions, as sketched in Figure 7.

### 5.1 Higher Power or Pulse Energy

Elevated output power or pulse energy serve as prerequisites for long‐detection‐range of LiDAR. Future research will continue to push the limits of output power or pulse energy, while addressing critical challenges including thermal management and mode purity degradation under high‐power.

### 5.2 Diverse Tailored Modes

The orthogonality and infinity of OAM modes is the key of vortex beam research and applications. Pursuing higher OAM order and on‐demand tailoring of multimode OAM modes, that is, the high‐dimensional OAM comb, are a critical direction for enhancing detection capabilities. Furthermore, family of sophisticated structured light fields, including vectorial vortex beams, optical skyrmions, and other topological beams, represent a critical tool for inspiring more novel applications. Therefore, developing superior intra‐cavity solutions for precise, efficient, and flexible tailoring of more complex structured beams becomes imperative.

### 5.3 Narrower Linewidth

Currently, LiDAR research based on the RDE primarily employs continuous‐wave lasers and focuses on detecting the angular motion of objects. However, in practical applications, there is a critical need for a multi‐dimensional detection LiDAR system capable of simultaneously acquiring information such as position, angular velocity, and linear velocity, as well as maintaining strong interference resistance. Both ToF for measuring distance and the linear Doppler shift for determining linear velocity impose stringent requirements on laser linewidth. Therefore, pulsed vortex lasers represent an essential pathway toward realizing such multi‐dimensional detection.

### 5.4 Device Integration

Device integration is a necessary path from laboratory principles to engineering applications. Recent years have already witnessed a progression from bulk solid‐state laser systems to integrated optical modules like NPRO and microchip lasers. In the future, solid‐state lasers are anticipated to continue their miniaturization trajectory toward chip‐scale integration. This evolution demands a continuously deepening understanding of mechanism of vortex lasers, coupled with knowledge from other disciplines and engineering fields, to persistently advance the development of integrated devices.

Solid‐state vortex laser at the eye‐safe band is currently at a critical stage of transitioning from fundamental research to engineering applications. With the continuous emergence of new materials, mechanisms, and technologies, greater breakthroughs will be achieved in terms of power, mode control (both transverse and longitudinal modes), linewidth, and integration. This will undoubtedly give rise to revolutionary technologies and applications in fields as LiDAR, and also other applications such as large‐capacity communication, imaging, laser processing, and quantum information.

## Author Contributions

**Haojun Zhang:** writing – review and editing, writing – original draft, investigation, visualization. **Zhichao Zhang:** investigation, writing – review and editing. **Lihong Hu:** investigation, writing – original draft. **Chunqing Gao:** supervision, writing – review and editing. **Shiyao Fu:** writing – review and editing, supervision, project administration, funding acquisition.

## Funding

This work was supported by the National Natural Science Foundation of China (Grants 62375014, 62550001, 62350011), National Key Research and Development Program of China (Grant 2022YFB3607700), and Beijing Natural Science Foundation (Grant 1232031).

## Conflicts of Interest

The authors declare no conflicts of interest.

## Data Availability

The authors have nothing to report.
